# Understanding Value Change in the Energy Transition: Exploring the Perspective of Original Institutional Economics

**DOI:** 10.1007/s11948-022-00403-3

**Published:** 2022-11-10

**Authors:** Aad Correljé, Udo Pesch, Eefje Cuppen

**Affiliations:** 1 Department of Values, Technology and Innovation/Economics of Technology and Innovation, Delft University of Technology, Jaffalaan 5, 2628 BX Delft, The Netherlands; 2 Department of Values, Technology and Innovation/Ethics and Philosophy of Technology, Faculty of Technology, Policy and Management, Delft University of Technology, Delft, The Netherlands; 3 Faculty of Governance and Global Affairs, Institute of Public Administration/Governance of Sustainability, Leiden University, Leiden, The Netherlands

**Keywords:** Value change, Institution, Energy transition, Technology, Transaction, Behavior

## Abstract

In this paper, we take inspiration from original institutional economics (OIE) as an approach to study value change within the highly complex assembly of sociotechnical transformations that make up the energy transition. OIE is examined here as a suitable perspective, as it combines Dewey’s pragmatist philosophy and a methodological interactionist perspective on value change, behavior and institutions, with technology figuring as a transformational factor. This combination overcomes conceptual and methodological shortcomings of alternative accounts of values. We will present the contours of an OIE based conceptual framework connecting nature, humans, technology, the economic process, society, culture and institutions and habits, valuation and behavior. We illustrate how to use this framework to examine and understand how environmental, ecologic, safety, economic, and social concerns about the energy transition are (re)framed as (new) values in the belief systems and habits of individuals and groups. Moreover, we will explore how that may give rise to collective action, via the institutionalization of such revised values in the procedures, arrangements, norms and incentives guiding transactions. As such, this approach allows us in a fine-grained manner to conceptually and theoretically understand the way in which values change in the energy-transition, as a complex interaction of technology development and social relations.

## Introduction

The ongoing energy transition not only entails the development and implementation of new technologies to produce, distribute and consume energy; it also brings about changes in societal practices. These changes include institutional adjustments for the implementation, operation and funding of sustainable technological solutions. Moreover, as changes in energy supply are highly pervasive in domestic, economic and social life, new experiences will induce new (positive and negative) perceptions. These, in turn, will influence (or arouse) values and may inspire collective action for adjustment; either in the choice or design of technology and/or in institutions. It is important to realize that the energy transition cannot be seen as a homogenous development, however. It is the conceptual aggregation of a wide array of attempts to change parts of the energy system. In this paper, we will make a cautious effort to understand and conceptualize this process by using insights derived from the approach of original institutional economics (OIE).

As an example one may think of the consequences of constructing wind farms to mitigate the threat of climate change by generating electricity without CO_2_ emissions. The intermittent character of the power produced by these farms has an effect on the operation of the electricity system, which may influence the conditions under which power is supplied to households and their modes of consumption. Moreover, the spatial planning of wind farms may give rise to local protest because of their visual and audible nuisance. Also debates may arise about the distribution of the revenues among the private investors and local communities; the latter falling victim to the nuisances. Such controversies will affect the trust in authorities and citizens’ and businesses ideas about the fairness of decision-making procedures (Pesch et al., 2017; Cuppen et al., 2020).

All these issues are the result of, or invoke, normative evaluations revolving around the moral question of how changes in sociotechnical systems relate to particular understandings of values, which, as a consequence, may also be subject to change (van de Poel, 2018; van de Poel & Taebi, 2022). We observe that a framework that allows for a coherent analysis of value change and the way in which humans deal with moral issues, as regards their relations with nature, technology, and institutions, in the context of the economic process, and within a particular society and culture may be helpful. We propose that OIE allows for the construction of a comprehensive framework that is helpful to study *changing moral evaluations* in a context of *technical and institutional change*.

The label ‘economic’ might raise some eyebrows among scholars in interpretative research traditions, given the reductionist nature of most economic theory. Yet, it is important to stress that OIE takes a different perspective than the mainstream school of economics, neoclassical economics, (NCE) and new institutional economics (NIE), and to a large extent aligns with social constructivist approaches. NCE theory engages with the question of how aggregated individual utility and profit driven motivations and actions of actors gives rise to particular allocative outcomes at the collective level. NIE introduces ‘bounded rationality’ as an important feature of these actors and highlights the role of institutions, as the *rules of the game*, in ordering and coordinating the transactions taking place, given their characteristics and the context in which they take place. Both approaches assume actors’ ‘preferences’ as given and exogenous to the analysis of the economic process of competitive optimization.

In contrast, OIE aspires to ‘understand’ (economic) behaviour as being part of a dynamic socio-technical processes, focussing on the continuous adjustment of individuals’ normative evaluations. It helps to understand the complexity of ‘managing’ the energy transition, by enquiring how these social values are changing and to what effect. As regards the energy transition, socio-technical change implies that we have to consider the interaction between advances in technology, the relationship with nature and natural resources, the economic process in which humans deal with nature and technology guided by institutions, and the way in which individuals and society deal with social values. Static ‘models’ drawing on fixed individualistic preferences are not appropriate here.

In the next section we will introduce the perspective on values as developed within OIE. Following pragmatist philosophy, values will be portrayed as meanings that allow for the normative understanding of social reality, as well as the wilful organization of that social reality. These values are reproduced within institutions that coordinate social interactions and guide individual choices. Experiencing these social interactions, actors will review the values they maintain, which possibly may give rise to a reconsideration of the institutions. In section three, the contours of an OIE-based framework, will be sketched. Having this initial framework will help us to understand how social change occurs and how such changes are interrelated with patterns of value change. Section four will analyze the energy transition, sketchily applying the framework. Section five concludes and presents a research agenda to further develop the OIE framework in relation to value change and energy transition.

## OIE and Values

OIE understands values as dynamic, holistic and systemic entities. With that, OIE takes distance from accounts that regard values as fixed given ‘preferences’ or ‘inclinations’ that guide human behavior, like in standard economics (see Wilber & Harrison, 1978) or social psychology (see Schwarz & Bilsky, 1987; Steg & De Groot, 2012). OIE also allows us to qualify the understandings of values developed in ethics and in social research, which are surprisingly opposed to each other. Ethical perspectives often assume values as moral entities that can be stripped from contingent influences, and that as such cannot be subjected to change (see Frankena, 1951; Korsgaard, 2015). Social research, in contrast, tends to depict values as collective agreements on certain goals and norms that are current within a certain cultural or institutional context (see Hitlin & Piliavin, 2004; Morris, 2017). This suggest that ethics appear to *underplay* the role of contingency in the establishment of values, while social science research such as Socio Technical Systems (STS) theory may *overestimate* contingency reducing them to functional outcomes of socio-political interactions.

The approach of OIE towards values should be seen as a *hermeneutic* approach, which is very much embedded in the pragmatist philosophy of John Dewey, as a highly important inspiration for OIE scholars. This implies that values are informing the understandings that individual people maintain and allow them deal with complex social phenomena; enabling them to take decisions anticipating future events (Dewey, 1922, 1939). As such, humans are seen as beings that constantly make judgements about what to do next, by interpreting situations in terms of being desirable or undesirable (Pesch, 2020). These normative interpretations are constructed and reproduced in social interactions.

In short, values provide humans with a higher-order categorization of meanings. They give normative significance to a wider range of experiences and projections, *aggregating* a variety of impressions, that allow agents to prepare for future actions (see Boenink & Kudina, 2020; Pesch, 2022). Values can be made explicit and then they can figure as reflective starting points for processes of collective deliberation and decision-making. They allow us to express and evaluate the desirability of certain future options and to intervene when necessary. In turn this enables us not only to think about pursuing consistent individual preferences, but it also allows us to *deliberate about the organization of society*, facilitating discussions about collective courses of action. In other words, the explication of values and the deliberation on their prioritization may support effective and legitimate forms of collective decision making.

As a school in economics, the focus of OIE is on interactions taking place in systems of production and consumption of goods and services, which often are based on technical forms of mediation (Mayhew, 2018). According to OIE scholars, values are developed and redeveloped by individual agents in such societal practices. These values are continuously adapted to changing contexts, building on previous experiences and in anticipation of expectations. OIE presents actors as habitually (inter)acting in an institutional structure which is continuously (re)created by their acting, confirming their ‘expectations’ as regards the ‘outcomes’. Yet, ongoing technical and societal changes, as well as the formation of new expectations about future developments may trigger individuals to reconsider their normative evaluations, potentially giving rise to value change at the collective level. This is especially relevant with regards to the energy transition, as this is an inherently future-oriented phenomenon, fed to a large extent by science-based outlooks and predictions about future developments, as well as promises about alternative energy technologies. This perspective is recognizable in the work of ‘classical’ original institutional economists like, for example, Veblen (), Mitchell (1927), Commons (1931, 1934), Ayres (1944, 1961), Zimmerman (1951), Foster (1981), their followers in the 1970s and 1980s, like Tool (1977, 1993, 1995), Bush (1987, 2008, 2009) Stevenson (1987), De Gregori (1974, 1977, 1987) and more recent scholars, like Hodgson (1998), Mayhew (1981, 2010, 2018), Lawson (2009, 2015), Bromley (2008, 2015), Groenewegen et al., (1995, 2021), and Vatn (2007).

As the name of OIE already indicates, *institutions* play a major role in this approach. Commons defined institutions as “collective action in restraint, liberation, and expansion of individual action” (Commons, 1934: 73). Douglas North (1991) stated that “Institutions are the humanly devised constraints that structure political, economic, and social interaction. They consist of both informal constraints (sanctions, taboos, customs, traditions, and codes of conduct), and formal rules (constitutions, laws, property rights)”.

The normative evaluations of individuals are always embedded within a particular social context, involving institutions. As part of these institutions, the normative evaluations of individuals give rise to collective norms, so that expectations become shared among a collective of individuals, implying that institutions come to coordinate the interactions between individuals. With that, OIE invokes a methodological interactionist perspective on values, behavior and institutions (see Groenewegen, 2021). In this perspective, all values, individual as well as collective, are constituted in interaction with each other. They can be either emergent or established but—most importantly—they can always be subjected to re-valuation, being judged and deliberated in a specific context of time and place.

OIE has brought together the notions about values in the so-called ‘social theory of value’. Taking distance from conventional conceptions of ‘rationality’ in economic theories, OIE sees individuals as ‘rational’ in the sense that they are able to exhibit “a concern for the well-being of the community as a whole” (see Bush, 2009 p. 301). This implies that, to make normative judgments in terms of value, individuals must be able to reflect on the way an action or decision has wider societal consequences. To this end, Bush (2009) distinguishes *values*, as providing the standards of judgment in use; the process of *valuation*, which is the application of those standards by individuals and society to determine whether an action is right or wrong; and the *value judgement*, which pertains to the questioning and deliberated choice of a particular standard to be applied for appraising a certain value.

As regards processes of *valuation*, these take place routinely and continuously, putting a value on an action without conscious awareness of the standard of judgment upon which they are based. Such valuations are based on cumulative experience, in which traditions and habits prevail. In contrast, *value judgements* ask whether the right (conceptualization of a) value is applied. A revised appraisal, then, may initiate changes in habits of thought and behavior, as it involves a rational and explicit reconsideration in selecting new standards of judgment (values). This demands an argumentation and prioritization of the most appropriate standards in making a particular valuation.

These normative judgments are regularly made by individuals and collectives to test the fit between values and situations, but they are especially relevant within the context of changing circumstances. New phenomena in empirical reality call for the need to reconsider values; the normative categorizations that we apply to interpret the world around us may not appear to be so useful any longer. To understand the world and to (re-)act in a meaningful way, values always have to be open to change.

In OIE, quite some effort has been made to distinguish different types of values. As from its genesis in the early twentieth century onwards, the ‘Veblenian dichotomy’ has presented two fundamentally different societal modes of valuation; *instrumental* versus *ceremonial* valuation. In the formulation of Bush (2009 p. 302), *instrumental valuation* is associated with the technological process of ‘tool use and skills’, correlating human behavior with the scientific understanding and mastery of causal processes with which the community must cope in provisioning itself and improving its capacity for 'associated living’ (according to Dewey). Instrumentally warranted values are subject to constant re-examination in the problem solving processes in society; testing the ‘instrumental efficiency’ in achieving the ‘ends in view’, reflecting the capacity for critical thinking that gives rise to, and is impelled by, technological innovation.

Secondly, there is *ceremonial* valuation which opposes the incorporation of new technology and institutions that may threaten the prevailing social relations. *Ceremonial values* are values that rationalize traditional patterns of power and status and rituals of the community, manifest in habitual patterns of thought and behavior, established by ideological preconceptions, myths, folktales, which are considered not subject to critical examination. The ‘test’ of ceremonial practice is ceremonial *adequacy*, as accorded by ‘influencers’ with charismatic powers. Early OIE scholars, like Veblen and Ayres, were quite convinced of the juxtaposed nature of identifiable instances of ‘good’ instrumental progress and ‘bad’ ceremonial resistance. Given the tensions between the old values and newly evolving technological realities, either old values are to be adapted, or technological, economic and societal progress will stagnate (Ayres, 1961; Bush, 1987, 2009; Samuels, 1977; Veblen, 1914).

Others like J.R. Commons, inspired by Dewey, pragmatically sought the answers in the ‘right’ societal processes and structures for deliberation. In a continuing debate, later scholars have either taken sides, or argued that a both modes of valuation go together. Indeed, the notion of ‘reasonable’ value, drawing on Dewey and Commons and further advanced by more recent authors like Bromley (2008, 2015) and Vatn (2007) is less stringent in distinguishing and juxtaposing instrumental and ceremonial values (see Ramstad, 1989, 1991). Indeed, in the end, value change will pertain to both ceremonial and instrumental values, so we can follow this more relaxed approach to the types of values, taking inspiration from OIE instead of seeing it as a dogma.

## Towards an OIE Framework for Understanding Value Change in the Energy Transition

OIE, as it has evolved over time, can be seen as a collection of fairly different approaches in economic analysis. It is often criticized for the lack of a consistent ‘theory’ (see Kaufman, 2007; Hodgson, 1998; Ramstad, 1995). We argue that addressing the issue of value change in the energy transition is not so much about providing a singular explanation or predictions drawing upon a unified theory, but in combining a selection of the relevant approaches in a consistent manner. OIE enables us to create an appropriate conceptual framework, that fits the issues at stake in understanding value change and the energy transition (see also Hodgson, 1998, 2004; Mayhew, 2018; Groenewegen et al., 1995; Groenewegen, 2021; van de Poel & Taebi, 2022). As will be outlined below, this framework will help to conceptually and empirically derive explanations for the occurrence of value change, as an interaction between different relevant ecological, technical, organizational, institutional and economic facets of the energy transition, and the consequences thereof.

**Fig. 1:**
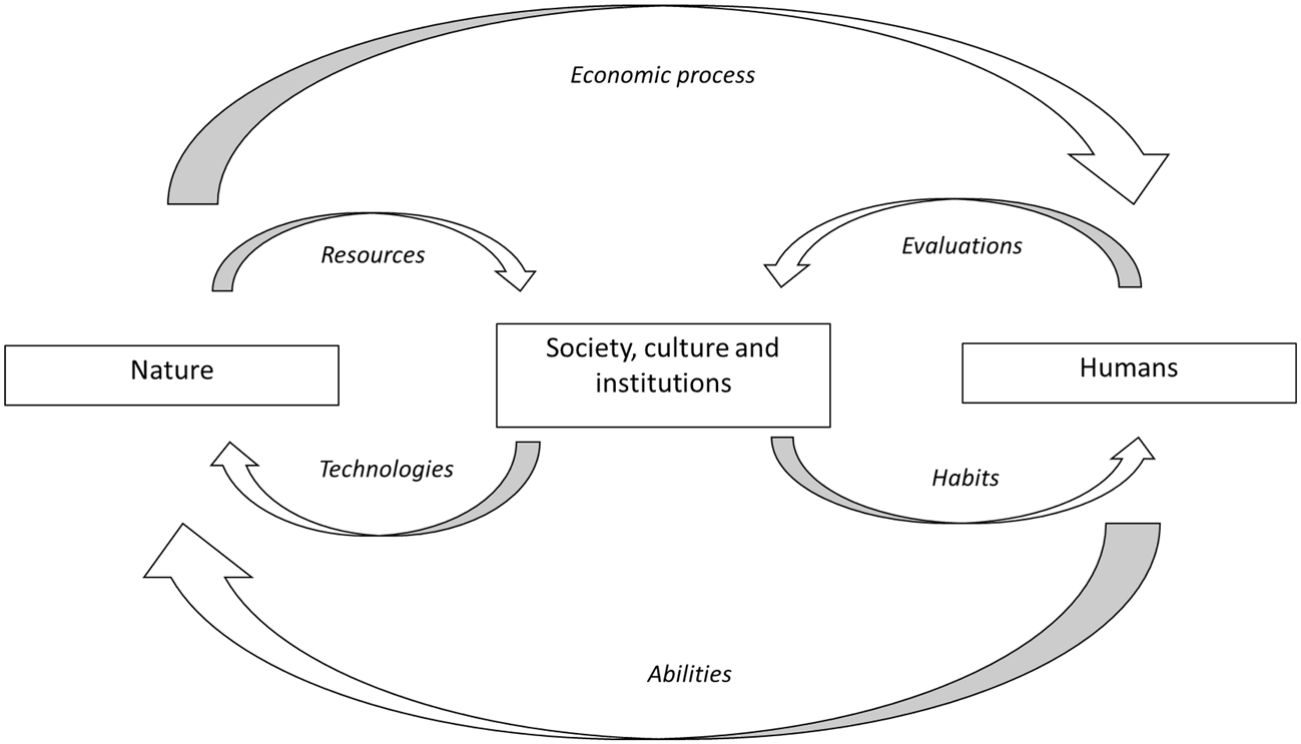
Humans in society handling nature

Indeed, we argue that the added value of applying an OIE perspective is that it allows us to connect a number of elements that are necessary for such an understanding. An overview of these ‘issues at stake’ is provided by Adkisson’s sketch (2010) of the main elements of an OIE perspective on economics and the role of values therein. The expansion thereof into our framework below is based on the simple notion that humans (have to) deal with nature, as is illustrated in Fig. 1. Humans as individuals possess some physical and cognitive abilities to handle ‘nature’. Yet, they will do that together with others, being part of (a) society, involving a particular culture and a set of institutions. The collective and coordinated use of human abilities to manage a variety of natural phenomena appears as a set of technologies. These are applied in the economic process of providing society with resources and necessities. Yet, the dynamic and expanding nature of technology also enhances human awareness of environmental and other effects of the economic process, providing insight in possible causes of these effects and potential solutions. Such awareness has an impact in the choices for ‘using’ particular natural phenomena and turning them from neutral stuff into ‘natural resources’ by employing particular technologies.

How society values the results thereof as acceptable (or not) is an outcome of the interaction of what is endorsed by the prevailing culture and the institutional setting, and the way individuals (in society) are able to derive, change and articulate their particular values. Indeed, humans are embedded in society which transfers habits onto them, while the act of (re-)evaluation allows for the adjustment of these habits. This interaction eventually determines how (changed) values are (either or not) translated into institutionalized public values and generalized individual habits of ‘how to do something’. And here also lie the impetus and rewards, both for society and its individual members, to develop new abilities and technologies ‘to do something differently’. In paragraphs below we will go into some detail in explaining the interaction between: (1) nature, humans and technology; (3) technology in the economic process; (4) society, culture and institutions; (5) habits, valuation and human behavior.

### Nature, Humans and Technology

OIE sees the human species as part of nature. Yet, unlike most other species, the evolution of the humans has provided them with the ability to ‘use the gifts of nature’ by physical and socio/cultural means (see Zimmerman, 1951; De Gregori, 1987; Moran, 2000). By using tools and transferable knowledge *as technology*, humans are able to understand, adapt to and manipulate their natural and physical environment, to some extent. Technology acts as a crucial mediating and transformative factor in interactions with the natural environment and with other humans.

Technology is seen as the combination of the mastery of physics and materials, and the ability to transfer the associated ideas and knowledge among people. Indeed, the creation and ‘use’ of technology, as tools and transferable knowledge, demands collaboration and is facilitated by communication and ‘working rules’ (see Ayres, 1944, 1961; De Gregori, 1977; Lawson, 2009). Essential is the notion that technology involves a problem-solving, evolutionary and accumulative process, in which the ongoing, more or less successful, (re)combination of previous technical solutions creates new ones. This also involves the understanding and evaluation of the positive and negative effects as the results of such innovative technology combinations in the economic process. Therefore technological progress is both cumulative, but also (potentially) reflective, as an ongoing social process.

### Technology in the Economic Process

OIE sees the economic process as the ‘system’ by which human beings collectively provide themselves with natural resources, harvested from the ‘gifts of nature’, to be converted into usable products and services, using technology. Zimmermann (1951 p. 15) elegantly stated: “Resources are not, they become; they are not static but expand and contract in response to human wants and human actions”. Such actions include the protection against the harshness of nature by all kinds of means, the creation of places to live and to harvest, and activities to convert natural resources into goods and services to ‘consume’ (see Ayres, 1961).

Zimmerman also stressed that “the word resource does not refer to a thing or a substance but to a function which a thing or a substance may perform, or to an operation in which it may take part […] attaining a given end such as satisfying a want” (Zimmermann, 1951 p. 7). Crucially important is that knowledge has a dual role in this process: “Resources are dynamic not only in response to increased knowledge, improved arts, expanding science, but also to changing individual wants and social objectives. [R]esources must reflect every change in the purpose of the appraiser” (Zimmermann, 1951 p. 11). This implies that in the economic process humans (may) exhibit a strong tendency to keep improving their situation, drawing on their ability to reflect on their previous achievements and to think out new ‘opportunities’ and overcoming perceived ‘resistances’.

It is in this ongoing process of appraisal, that values are applied as standards of judgment, to determine whether such achievements are to be considered right or wrong. This requires a continuous interaction of individuals and groups in social deliberation, as to whether the economic process yields what is considered necessary and desired. The direction of this process is dependent on culture; namely, the ability to communicate and deliberate, given human attitudes and relations, and to evaluate what natural environment and resources are perceived as ‘wanted’ (Brinkman, 1997; Mayhew, 2010, 2018).

### Society, Culture and Institutions

Hayden (1988) explains that it is in the notion of culture that technology and the (in)ability to communicate, collaborate and reflect come together. Hayden sees (a) culture as a particular combination of material expressions and evident norms (beliefs and attitudes) regularly expressed or implicitly held.

As stated, OIE considers society and the economy as a system in which a) people engage in all kinds of activities to support and enjoy their life on earth, by making use of increasingly ‘effective’ technologies they develop and learn to master. They find ways to culturally organize and to cooperate in employing these technologies in societal and economic structures. This leads to all kinds of institutionalized ‘going concerns’ (like firms, societal and public organizations, etc.) of a different purpose and nature (political, economic, social, cultural) to organize the provision of goods, services and governance to society. This happens by means of a multitude of transactions within and between these going concerns and their participants (see Commons, 1934).

In a more or less hierarchical setting, the individual ‘members’ of these going concerns act, react, follow, initiate and choose. In doing so, these actors are constrained and enabled by formal and informal institutions, involving values associated with the going concerns in which they are embedded, as well as by their own habits or ‘mental maps’ (see also Ayres, 1944, 1961). Both these institutions and actors’ individual habits provide a top-down explanation of how individual people and groups are guided, within and through their complex socio-technical environment (see Bush, 2008). So, to understand the behavior and role of individual and collective actors within their going concerns, as well as the occurrence of institutional change, it is essential to analyze the *recreation and the creation* of underlying (patterns of) values.

### Habits, Evaluation and Behavior

From the bottom-up perspective of the individual human being habits, instincts and customs are seen as important drivers and motivations for behavior and decision-making. Habits (and instincts) are dispositions of actors that have evolved over periods of time and reflect past beliefs and experiences. Yet, habits are not just involving mechanically repeated behavior. This is because human actors are also ‘volitional’; they have more or less capacity to deliberate and to choose, depending on their environment (Bromley, 2008, 2015; Commons, 1934).

This means that people are reflective in respect of what ‘is’ and how it ‘ought to be’. They are capable of evaluating ‘the way things are working’ because of their ability to understand and evaluate the characteristics and quality of their life in their natural, ecological and social environment. This ability results from the use of insights drawing upon (newly developed) technologies to observe, measure, bundle, convey, compare, or influence, societal and environmental ‘trends’. Obviously, also the capacity to communicate about that with others derives from technology development in transport, printing books, mass media and, currently, the social media.

In such a world with an ever-expanding capacity (and need) to reflect, OIE sees actors as being able to identify habits, to analyze how these influence behavior and to evaluate whether the habits as such contribute to realizing the desired consequences of actions, or not. Generally, it can be stated that stability and acceptance will prevail, as long as ‘going concerns’ go on without disturbing effects in terms of ecological, economic or social consequences, and when there are no potentially disruptive challenging technical or organizational innovations and better alternatives at hand. If disruptive effects arise, however, then actors can make the existing habits and institutions and their consequences explicit, and start a process of collective deliberation. In an attempt to change these habits, they may engage in the search for new standards of judgement for pre-existing values, or even for new attributes to be encapsulated by a value appropriately (Bromley, 2008, 2015; Commons, 1934; Hodgson, 2004).

Dewey (1922, p. 28), explains that habits can be inquired and tested by man. Actors can take distance from the specific habits that cause an action and reflect on the consequences of that action. Normative propositions, Dewey says, “are capable of being tested by observation of results actually attained as compared with those intended” (1939, p. 24). Indeed, in the case of routines man acts mechanically, without thought about the consequences and without valuation of the consequences of the routinized actions in the light of the societal goals. When such reflections raise doubts about the rightfulness, or desirability of these consequences, then humans are in the position to inquire what is wrong about the habits. Then they may engage in collective action to intervene by altering the institutions (the rules of the game) to change the ‘habit of thought’. Hence, the real opposition is not between reason and habits, but between reasonable habits and unintelligently routinized habit (Costa et al., 2011).

With these elements, patterns of societal change can be explained in a clear way. Most saliently we can think of the way in which institutional changes in the economic process incentivizes new technological development, so that entrepreneurs gain competitive advantage (Pesch, 2018; Schumpeter, 2000). This framework also shows that voluntary change may be pursued, for instance when the undesirability of certain behaviors become manifest. This does not mean that voluntary change occurs without any effort, but it makes clear that due to changing values, attempts to change individual behavior or collective institutions may be instigated. What emerges here is a dynamic picture of social reality in which normative evaluations are transformed into behavior and institutions that secure these normative evaluations to become values with relatively stable character. Nevertheless, these values can always be taken into reconsideration, especially due to technological and other changes in society that demand normative re-evaluation so to adjust behavior and institutions. Such a normative re-evaluation can take shape as the introduction of new values, the reordering of values, or a modified understanding of values.

## Value Change in the Energy Transition

Dewey’s pragmatism, in combination with the OIE focus on the dynamic interaction between humans, nature and technology, in the context of the economic process, society, culture and institutions and habits, provide the ingredients for a framework for understanding, explaining and discussing the process of valuation, value change and the resulting behavior in the energy transition (see Bush, 2008; Vatn, 2007).

Indeed, this transition, as a fundamental reordering of the ways to produce, distribute and consume energy, brings about the demand for a reconsideration of the habits and values traditionally connected to human behaviour in the energy domain. Obviously, the need for this transition is instigated by the technological and scientifically derived insight that burning fossil fuels is emitting CO_2_, which causes global warming with malicious consequences. This drives the emergence of new ecological values in the process of valuation in respect of the functioning of the existing energy system, resulting in the need for a future energy system without CO_2_ emissions.

This objective motivated experts, engineers and political decision-makers to set off a fundamental socio-technical transformation of the energy system. Of course, in the 1960s and 1970s the rise of environmentalism also gave rise to new ecological values, suggesting limits to sulphur, toxic metals and fine particles emissions and inspiring political action and policy to that end. Quite saliently, these re-evaluations have given rise to innovation and *technical* solutions, involving shifts in the use of primary energy resources away from coal and heavy fuel oil, and the wide-spread application of filters and other devices in the end-use of energy, like in boilers, car engines and heating appliances.

In the current energy transition in response to the need to reduce the CO_2_ emissions, the technical focus is also dominant in suggesting shifts in the production of new primary energy resources (like solar, wind and geothermal energy), the conversion of these resources into end-use energy carriers (like hydrogen), the means of transport (like district heating), and the use of different and new appliances, like the heat pump, low temperature heating and led illumination. A more or less hypothetical perspective that arises from a wide range of academic, consulting and industry publications suggests the following changes to the energy system:

- The shift from fossil based primary energy resources towards renewable and low CO_2_ resources, like wind, solar, geothermal, bio gas, and a variety of other sources of energy;
- The shift from a predominantly centralized, large-scale, production of end-use energy carriers, towards a geographically dispersed pattern of production in units, ranging from large scale off-shore wind parks, to local biogas and geothermal facilities, and the domestic production of electricity or hot water energy with roof top panels;
- A shift from a one-directional transport system, from production to conversion and end-use, towards a bi-directional system enabling the production, storage or conversion of energy at several levels, from large scale central to domestic systems.
- The shift from a system with a limited availability of information as regards the use and production of energy, towards an ICT-driven system in which this information is immediately available, directly linked to systems users and their equipment and appliances.
- The shift from an essentially demand-driven supply of end-use energy, towards a ICT-driven system in which the use of energy (or the demand) is made responsive to the availability or scarcity of end-use energy, by demand side management and price-driven incentives for energy consumption;
- A more prominent focus on energy savings and efficiency by insulation and advanced appliances in the end-use of energy in households, businesses and industry.

These technological shifts figure as points of orientation in the transition for many scholars, and for technology developers, businesses and decision-makers, in the search for effective public policy and incentive structures to stimulate the transition. This technical framing seemingly allows for other societal values, such as comfort, access, security of supply, safety, affordability and economic welfare to remain unaltered (also see Rodhouse et al., 2022). However, it is becoming clear that these technical changes cannot be achieved without concomitant societal changes. It is in understanding what these societal changes will bring about that the notions of value change as derived from the OIE literature are helpful. Below, we will provide the contours of the way in which these changes are likely to occur. Further research is needed to substantiate these changes, for the agenda-setting purpose of this paper, we will have to acknowledge the tentative character of this list.

To start with, in addition to technological change, the energy transition will also bring about a radical *institutional* transformation of the current energy system, as regards the coordinative mechanisms and transactions applied. In the traditional mono-directional mode the expected use of energy is translated into demands upon production, storage and transport facilities, to be honored via either market-driven or regulated supply commitments by producers and grid-operators. In the newly developing system, it is to be expected that there will be a much larger variation in the way in which end-users determine and arrange their use (and provision) of energy; either by using less or more energy and/or by producing their own energy, or buying their requirements. Depending on the ‘arrangements in place’, this may create situations in which both users as well as producers, or combinations thereof, contractually delegate part of their control over energy use or supply to other (aggregating) actors in the system. This has important implications in terms of the individual and public values covered in property rights, contractual arrangements and in associated information flows, the investments and financial cost, and eventually the degrees of freedom and decision rights in producing and using energy for particular purposes (See Sioshansi, 2020; Glachant et al., 2021).

This technical and institutional transformation may influence the *habitual practices* of energy suppliers and/or energy users (or prosumers), which in essence comes down to the emergence of new ways of transacting. For *consumers* this transformation will imply that—in their habits—they are confronted with a relatively new ‘problem’ in valuing, and determining whether and when to use what kind of energy and for what purpose; whether to purchase or produce or to store it; what appliances to buy, and with whom to engage under what kind of conditions. Obviously, they may contract out such decision-making to other trusted parties that select the arrangements on their behalf. In some locations, the choice for a particular form of energy supply and associated appliances will be mandatory, for example when they are serviced by low-temperature geothermal district heating systems. This, obviously, will have a large impact in the habits of their daily life, as many aspects of that life (in the past and today), depend on the availability of the right forms of energy at the right time and the appliances to be used (See Weijnen et al., 2021).

For *producers and other supplying actors* it implies that they will have to decide with whom to contract, to buy, process and sell energy, or provide services or appliances, and under what conditions. For *public authorities*, like for example municipalities and grid operators, it raises questions as regards their role(s) vis a vis citizens and businesses, responsibilities and interventions in the system and in the governance of the transactions involves. Main issues involve the pursuance of specific energy supply and consumption objectives, the linkage between decision-making in respect of spatial planning, transport and the built environment and the system of energy provision and the nature and form of public values they should honor in the provision energy to their inhabitants as a public good (See Weijnen & Correljé, 2021).

Considering these changes, the transition will have implications in the process of valuation. This concerns, as argued above, the choice and the nature of the specific issues to be valued and the values to be applied (i.e. the judgement). It is also about the ‘migration’ of individual values into the collective domain via individual and collective forms of *decision-making*, either by consumers, producers, or in the public realm. To be sure, in the variety of transactions associated with the transition, new actors with ‘new’ values will emerge. For example, passive consumers will turn into reactive prosumers possibly linking up into collectives. Local energy systems may emerge operating (partly) in isolation from the central system. New digital flows of new information, collected and processed by ‘aggregators’, will facilitate the creation of novel property rights on energy flows, stocks and capacities in the system and time slots. These rights (and liabilities) will either or not be made available to actors, or forced upon them, by energy service providers or municipalities (See Weijnen & Correljé, 2021).

All of these (new) transactions constituting the energy transformation raise questions about the values at stake and the way in which these are (to be) dealt with. These values are incorporated in a variety of formal institutional structures, like laws, norms, standards, regulations, environmental impact assessments, permits, and private contracts, and in informal institutional arrangements, like customs, traditions, habits and routines. To be sure, the transformation will affect, positively and negatively, the collection of individual and public values currently sustained by (groups of) people, businesses and (public) authorities. And different patterns of value change will be observed; not only in the different energy end-use domains, i.e. domestic, industrial and transport, but also in industrial sectors providing electricity, gas and petroleum products. In this respect, system integration is an interesting phenomenon, in the sense that new forms of energy are introduced in particular end-use segments that were dominated previously by particular energy carriers. A case in point is the automotive sector, where the reliance on gasoline and diesel provided by the oil industry is being replaced by electricity. Therewith the values associated with automotive mobility get connected with those in the electricity sector and with our habits the domestic sphere, via smart power supply system at home.

The importance of identifying, describing and understanding these patterns of value change and examining the normative character of such changes is that it directs us towards the discussion about which society we want to have. The energy transition is not a force of nature, but a set of human choices that are made to create a more desirable society. The framing of the energy transition merely as a science-based and technically determined phenomenon tends to obscure that this transition is above all an endeavor that brings about a range of moral challenges at many different levels. Foregrounding these challenges allows for much more constructive forms of collective direction setting.

## Towards a Research Agenda: OIE and the Energy Transition

In our reading and exploration, OIE provides the ingredients that allow for a comprehensive analysis of value change in the ongoing energy transition. Indeed, the basic concepts that are necessary for such an analysis are present, connecting nature, humans, technology, the economic process, society, culture and institutions and habits, valuation and behavior.

We have picked ingredients from the long tradition of OIE. In this, we have been admittedly eclectic and brief, and a coherent framework that allows for the dedicated analysis of value change in the energy transition needs to be developed. To do so, a further examination of the energy transition in terms of potentially useful OIE-ingredients is necessary.

The energy transition cannot be seen as a homogenous development. Instead, it is the conceptual aggregation of a wide array of attempts to change parts of the energy system. These changes start from a variety of technical imaginaries that become more or less widely endorsed in different domains. These changes induce a wide range of more or less radical transformations, including the coordinative mechanisms that are embodied by institutions. This will influence the ways in which both ‘energy suppliers’ and ‘energy users’ (or prosumers) will experience the changes in their transactions and therewith in habitual patterns of energy supply and/or consumption.

The collective behavior of all the ‘users’ of the energy system will have an impact in the functioning of the system as a whole, and in the emerging organizational and technological patterns of energy production, supply and end-use. At the same time, however, the functioning of the energy system will have an impact on the values and behavior of the ‘users’. We underline that it is particularly in the face of potentially disruptive challenging technical or organizational innovations that actors can make the existing habits and institutions and their consequences explicit. The may start a process of collective deliberation in search for more appropriate standards of judgement for pre-existing values and new attributes to be addressed. This process of re-valuation will be of influence in actors’ habits and behaviour; how they use the system, how they will interact with new installations, infrastructures and appliances, new flows of information, new ‘mechanisms of coordination’ and other actors in the system, and to what extent they will accept such changes. A crucial question for research addresses the patterns that can be observed in (emerging) mechanisms of value change and how these facilitate reflection and deliberation.

Via mechanisms of collective action, either in the democratic political process, or ‘out on the streets’ and via social media, values are expressed and may drive the creation of new rules and institutional structures, which will coordinate the way in which transactions are shaped and (allowed to) take place. This, moreover, will happen in an emergent, dynamic manner; the system users will act, react and interact being motivated by their experiences over time and their individual and collective evaluation of these experiences. Here the important question should be addressed as to what kind of changes the emerging ‘energy transition’—as a dynamic process of interaction between the elements referred to above—will bring about in the mechanisms of collective action, political articulation and, democratic (or less so) decision making. This begs for the question as to what impact normative reflection or analysis may have on the different types of transactions undertaken by public entities, businesses and pro/consuming citizens.

Obviously, here, a critical issue is the way in which national, or even regional and local, traditions and culture of governance are of influence in these processes and their interaction. Indeed, both the need to scale-up the energy transition and the integration of the separate energy supply pillars, demand a better understanding of the emergence and impact of value change. This should also involve the recognition that a system of multi-level governance is developing in which the domestic sphere is connected in specific ways with the values and technicalities at the local and municipal, the regional, the national, the continental and the global level (Correljé, 2021; Scholten, 2018). Possibly, this is an issue that has received less attention in the OIE scholarship, and as such, it requires further development.

We see a clear perspective for an OIE–based framework to fruitfully contribute to research on the energy transition in several areas. These involve studies focusing on the scaling up of technology and the governance model, also involving the multilevel perspective (Geels, 2020), studies on public values and the energy transitions (Correlje et al., 2015; Demski et al., 2015; Ruef & Ejderyan, 2021, studies on energy practices and their relation to value change (Shove & Walker, 2014, Sahakian & Bertho, 2018) and studies on energy justice (Bouzarovski & Simcock, 2017; Jenkins et al., 2016, 2018; Sovacool & Dworkin, 2015; Sovacool & Dworkin, 2014, Sovacool, et al., 2016).

Admittedly, OIE sometimes comes with a terminology that is somewhat archaic. As such, we would welcome a terminological update of OIE and a more thorough comparison of this approach with analogous constructivist accounts. Also in other respects, there is still quite some theoretical and conceptual work to be done, to employ the insights of OIE.

The account of technology and its relationship to social and economic change of OIE is to a large extent reminiscent of ideas about technology endorsed in modern STS and innovation studies. However, by its inspiration on the pragmatist approach of Dewey and its focus on the way in which transactions are guided, structured, and facilitated, a la J.R. Commons, a more prominent and coherent approach to value change can be developed. Commons devoted ample attention to the study of the struggle between private and collective action in controlling, liberating and expanding individual action via public values in economic transactions, with strong focus on the US labor relations in the first half of the twentieth century. This work inspired his more general perspective on the organization of transactions and economic activity, upon which OIE was based. His systematic account of OIE regarding the way in which transactions emerge and how they are coordinated by public values incorporated in institutions as formal societal structures and informal beliefs and routines at several levels of governance, allows for a comprehensive understanding of value change in complex social phenomena. As such, OIE allows for a level of systematization that is often lacking in constructivist approaches. It is much more than a stale theory of the past; it may be revived to increase our command of the role of value change in societal developments such as the energy transition.

A final, and perhaps decisive, asset of OIE is that this approach not only allows values to be conceptualized as hermeneutic aspects that guide individual and collective behaviour, but also that these values can be made *explicit* and reflected upon (See also Correljé et al., 2014, 2015). The ‘social theory of value’ makes clear that processes of valuation and value judgment can be subjected to deliberation, if the culture enables that. We can actively and collectively debate about which values are to be prioritized and which valuations are to be applied in certain cases and contexts. The normative orientations of society are not necessarily established by authoritative actors, by traditions, or by folk beliefs. Instead they can be opened up for democratic debate (cf. Roeser & Pesch, 2016). Therewith, in concrete processes of transition in (local) systems of energy provision, an OIE-based approach enables a more explorative and rich discussion of the relevant values and their interpretation among stakeholders (see for example Rodhouse & Correljé, 2022). Indeed, knowing how values are shaped in the dynamic interplay between individuals, institutions, technologies and culture allows us to think about how these values *ought to be shaped*, allowing for *the creation* of an energy transition that is responsive to societal and moral demands.
